# Pivotal Studies for Drugs About to Be Launched for Rare Diseases: Will They Better Support Health Technology Assessment and Market Access than in the Past?

**DOI:** 10.3390/jmahp13030037

**Published:** 2025-07-25

**Authors:** Claudio Jommi, Marzia Bonfanti, Melissa Guardigni, Andrea Aiello, Andrea Marcellusi, Pier Luigi Canonico, Fulvio Luccini, Chiara Lucchetti

**Affiliations:** 1 Department of Pharmaceutical Sciences, Università del Piemonte Orientale, Largo Donegani 2, 28100 Novara, Italy; pierluigi.canonico@uniupo.it; 2 Pharmalex Italy SpA, Largo Guido Donegani 2, 20121 Milan, Italy; marzia.bonfanti@pharmalex.com (M.B.); melissa.guardigni@pharmalex.com (M.G.); andrea.aiello@pharmalex.com (A.A.); fulvio.luccini@pharmalex.com (F.L.); chiara.lucchetti@pharmalex.com (C.L.); 3 Department of Pharmaceutical Sciences, Università degli Studi di Milano, Via Luigi Mangiagalli 25, 20133 Milan, Italy; andrea.marcellusi@unimi.it

**Keywords:** medicines, rare diseases, pivotal studies, study design, assessment, market access

## Abstract

The designs of clinical trials of drugs for rare diseases are challenged by health technology assessment organisations and payers. Phase II pivotal studies, single-arm or open-label designs, the extensive use of non-final endpoints, and the limited use of patient-reported outcomes (PROs) are the main points of contention. The evidence on the actual design of these trials is limited, but corroborates the concerns of the above. Our aim is to scrutinise whether the design of pivotal studies of drugs for rare diseases to be launched into the Italian market by 2026 present similar issues. The drugs and the relevant pivotal studies were retrieved from Biomedtracker and US and European clinical trial databases. We identified 154 new drugs for rare diseases. Single-arm designs account for 36% of trials. Almost 50% of randomised control trials (RCTs) are designed using an active comparator and 61% are double-blinded. Primary endpoints are mostly (82%) surrogate. A total of 59% of studies include PROs. Our findings were partially expected (e.g., extensive use of surrogate endpoints) and partially not (e.g., RCTs and an active comparator), considering previous studies on the same topic. Having more head-to-head studies may reduce uncertainty concerning evidence at market launch, but different issues persist, including the still limited role of PROs.

## 1. Introduction

The assessment, appraisal, and price and reimbursement negotiation of drugs for rare diseases are complex for several reasons.

Data on rare diseases, especially those for which valid treatments are not available, are in general limited. Scarce information affects disease epidemiology and natural history, clinical pathways, treatments used—often off label—in clinical practice, as well as prognosis, disease severity, and economic burden. Furthermore, patients affected by a rare disease are often heterogenous, causing delayed or incorrect diagnoses that make it even more difficult to estimate incidence and prevalence [1]. Limited information may have an impact on the design of clinical trials due to the lack of consensus on relevant endpoints, comparator(s), and barriers to patient recruitment [2,3]. The absence of valid therapeutic alternatives, typical of drugs with orphan designation, on the one hand, represents a distinctive factor of unmet need, and therefore impacts the value of the drug; on the other hand, it can be challenged by health technology assessment (HTA) organisations and payers when drugs used off-label in clinical practice are identified [4].

Rare diseases may seriously affect the quality of life of patients. Patient-reported outcomes (PROs) are increasingly included in clinical trial design as secondary endpoints and used for marketing approval [5]. However, the identification of PROs for rare diseases is complex due to limited knowledge of the disease in question and patient heterogeneity. Disease-specific PROs for rare diseases are often not validated when clinical trials are designed and generic PROs may not be suitable for rare diseases and the relevant treatments [6]. Indeed, while disease-specific PROs are often validated and accepted for regulatory approval, their acceptance by HTA bodies is not guaranteed. A scoping review highlighted that disease-specific instruments, although sensitive to particular conditions, frequently lack the utility values required by some HTA bodies. This limitation can hinder their applicability in cost–utility analyses, which are central to many HTA evaluations [7]. Furthermore, a study analysing HTA appraisal reports from agencies in Canada, France, Germany, Scotland, and the UK found that, although PRO data were included in 77% of reimbursement submissions, many received unfavourable assessments. The primary reasons cited were the absence of predefined analyses for responders, the utilization of non-validated tools for collecting PROs, and uncertainty in PRO measurements and meaningful changes in scores [8].

Clinical trials of drugs for rare diseases are affected by regulation too. According to EU Regulation 141/2000, orphan medicines can be approved after clinical trial Phase II [9]. On one hand, this regulation allows for the accelerated approval of orphan drugs, expediting patient access. On the other hand, the evidence provided for comparative assessment and market access purposes is considered by HTA organisations and payers less robust.

The economic elements supporting the negotiation of price and reimbursement (P&R) are the consistency between the incremental benefit and cost (value for money/cost-effectiveness) and the impact on expenditure (budget impact analysis). The robustness of cost-effectiveness studies is affected by the uncertainties of clinical benefit, disease history, and patient journey. It has also been argued that traditional cost-effectiveness models may inherently disadvantage conditions with no existing treatment options. In such cases, the lower cost of the “do nothing” alternative, due to earlier mortality, can artificially inflate the incremental cost of new therapies [10]. Budget impact could be further impacted by the difficulty of detecting the dimension of the eligible population. In addition, threshold values for the incremental cost-effectiveness ratio (ICER) could be systematically overrun, since the price requested for orphan drugs is often higher than for other medicines. This could be managed by setting explicitly higher threshold values for orphan medicines, or allowing reimbursement to orphan drugs if the ICER is over the threshold, or using a multidimensional evaluation in which cost-effectiveness represents only one of the evaluation criteria. For example, in England, higher thresholds for ICER and multicriteria approaches are used for highly specialised technologies, including drugs for ultrarare diseases, and the mean ICER for approved orphan drugs is well above the threshold range [11].

The inadequacy of patient viewpoint has been also stigmatized by the literature, including poor consideration of the indirect costs, health-related quality of life (HRQOL), and disability-adjusted life years (DALY) [12,13].

Many contributions raised evidence gaps for drugs for rare diseases focusing on challenges posed by rare diseases [14], the identification of efficacy endpoints [3], and approaches to integrate efficacy and safety [15]. Less and out-of-date evidence is available on the actual evidence gap of trials [16]. Only a recent analysis of 87 pivotal clinical trials for new drugs with 76 orphan indications approved by the FDA from 2017 to 2023 highlighted poor quality designs (frequently involving no randomization, blinding, placebo, or no treatment control) [17].

In Italy, HTA and P&R negotiations for new medicines and indications are managed by the Italian Medicines Agency (AIFA) [18]. P&R negotiation is carried out using a multi-criteria approach: disease severity, the unmet need, the added therapeutic value, the cost of therapeutic alternatives, the size of the target population, the cost-effectiveness profile, and the drug and healthcare budget impact are considered [19]. Pharmaceutical companies may also apply for innovative status for each indication approved. An innovative drug benefits from a dedicated fund and immediate access to regional markets, which is very important due to the regionalised organisation of the Italian health care system. The innovativeness status is decided on the grounds of the unmet need, the therapeutic added value, and the quality of the evidence through the Grading of Recommendations Assessment, Development, and Evaluation (GRADE) method [20]. Drugs for rare diseases have the same HTA and P&R paths as other medicines. Companies are only allowed to submit a P&R dossier immediately after a CHMP positive opinion. The industry can also rely on higher flexibility in the management of the GRADE evaluation by AIFA: AIFA acknowledged that applying criteria used to downgrade clinical trials (phase II, single-arm or open-label, etc.) to medicines for rare diseases would imply a negative decision for many orphan medicines [20].

Our aim is to investigate whether the limited evidence is also affecting drugs for rare diseases about to be approved in the European Union and launched in Italy by 2026. More specifically, we aim to answer the following question: does the design of pivotal studies of drugs for rare diseases about to be launched into the market present similar issues to the ones scrutinized by previous studies, i.e., Phase II, single-arm or open-label randomised controlled trial (RCT), extensive use of non-final primary endpoints, limited use of PROs?

## 2. Materials and Methods

Our research relied on a descriptive analysis of trial designs for drugs for rare diseases and involved three phases: detecting drugs expected to be launched by 2026; retrieving trials for these medicines; analysing clinical trials to understand design robustness.

The analysis was conducted from an HTA perspective, i.e., looking at the typical criticisms of methodological robustness, generalizability, and the relevance of endpoints (including PROs).

New medicines for rare diseases and new rare indications were retrieved from the Biomedtracker database [21], where drug development is tracked through public sources, including company press releases, archives, medical conferences and journals, clinical trials, registries, and regulatory documents. With an extraction conducted on 16th November 2023, the database identified a list of drugs with more than a 70% likelihood of approval based on value at baseline (that depends on the target and the level of unmet need) and an analysis of the Biomedtracker rating, which mostly depends on trial data robustness. The 70% threshold was validated through consultation with national experts in drug evaluation, pricing, and market access. These experts provided insights based on historical trends, regulatory experience, and pipeline analysis.

This list was integrated using the following assumptions/data:

- we assumed that all drugs for rare diseases approved in Europe between 2022 and early 2023 will be available in Italy in 2024, with the median P&R approval time in Italy being 463 days [22];
- all drugs for rare diseases that have undergone the Biologics License Application (BLA) or New Drug Approval (NDA) procedure in the USA, with a probability of advancing to the next approval phase equal to or greater than 70%, were assumed to reach the Italian market in 2025 considering an approval time in Europe of 277 days [23];
- all drugs for rare diseases currently in Phase III, with a probability of advancing to the next approval phase equal to or greater than 70%, were assumed to reach the Italian market in 2026.

Clinical trials and the relevant information were derived from the Biomedtracker database [21], complemented by the US [24] and EU clinical trial databases [25]. We included only trials identified as pivotal, defined as those explicitly described as such by the sponsor or clearly designed to provide the main evidence of efficacy and safety for a marketing authorisation application expected by 2026. Although the majority of included studies were Phase III, Phase II/III and, in 18 cases, Phase I or I/II trials were also considered if they met the above criteria or no more advanced trials were available for the same indication.

From each trial we retrieved the information on drugs to identify specific clusters and clinical trial design, and to evaluate its consistency with HTA requirements as follows:

- Drug clusters:
  1. Orphan designation (yes/no). According to EU Legislation, to qualify for orphan designation, a medicine must be intended for the treatment, prevention, or diagnosis of a disease that is life-threatening or chronically debilitating. The prevalence of the disease in the EU should be not be more than 5 in 10,000 (rare disease) or it must be unlikely that the marketing of the medicine would generate sufficient returns to justify the investment needed for its development. Finally, no satisfactory method of diagnosis, prevention, or treatment of the condition concerned may have been already authorised, or, if such a method exists, the medicine must be of significant benefit to those affected by the condition [26];
  2. Drug for rare or ultra-rare diseases (the prevalence of the disease is not more than 2 in 100,000);
  3. Drug class (new molecular entity—NME/non-NME/biological);
  4. Accelerated procedures (breakthrough/fast track/priority medicines—PRIME).

- Study design:
  1. RCT/single-arm;
  2. Within RCTs, double-blind/open-label;
  3. Within RCTs, comparator type (active/placebo).

- Endpoints:
  1. Primary (overall survival/safety/surrogate endpoints);
  2. PROMs (patient-reported outcome measures) (disease-specific/generic/both).

## 3. Results

Table 1 illustrates the characteristics of the 154 drugs retrieved from the databases. 

Haematology, onco-haematology, and oncology account for 21%, 18%, and 16% of the total, respectively. Other rare diseases for which drugs are under development are in the metabolic (15%) and autoimmune/immunological (14%) areas. The drugs in the pipeline are 51% biological; 42% of the drugs are derived from chemical synthesis and qualified as NME, and 7% are not qualified as new molecules. Of the 154 drugs, 119 (77%) have orphan designations and 13 (8%) address ultra-rare diseases. More than 60% of the drugs were included in US/EU accelerated approval programmes.

Table 2 illustrates our findings on trials for rare drugs. Additional information is provided as Supplementary Material (Table S1). Data are reported as total, oncology, oncohaematology, and others. Oncological drugs are expected to benefit from more established endpoints, validated PROMs, and structured trial designs such as master protocols.

More than 80% of the trials are classified as Phase III studies.

Only 36% of the trials are designed as single-arm studies. Even for orphan medicines, RCTs are more common (62% vs. 71% for non-orphan medicines), whereas for ultra-rare drugs RCTs account for 54% of the 13 trials. Studies designed as RCTs are more common, in line with expectations, in Phase III and, with the exception of the haematological area, in all therapeutic areas (Supplementary Material, Table S1).

In 46% of RCTs the comparison was carried out against an active comparator (Table 2) and no difference was found between orphan drugs (47%) and others (44%). The use of an active comparator is prevalent in onco-haematology (95% of RCTs) and oncology (56%). The use of active comparators in the control arm prevails in Phase III RCTs, biological drugs, and ultra-rare diseases (for which data, however, are affected by a very small number of observations) (Supplementary Material, Table S1).

A prevalence of double-blind studies (61%) is observed compared to open-label studies (Table 2). Open-label studies are more common only for biological drugs. This result is possibly caused by using oral treatment as a comparator that makes blinding not possible, since biologics are parenterally administered (Supplementary Material, Table S1).

Surrogate indicators are the most used primary endpoints in clinical trials (82% of the total), whereas overall survival accounts for 10% of the primary endpoints used (Table 2). Surrogate indicators prevail as the primary endpoints for all drug clusters. Overall survival was selected as an endpoint only in Phase III trials, was more frequently used (36% of studies) in oncology studies, and was almost completely absent in the study of drugs undergoing accelerated approval (Table 2 and Supplementary Material, Table S1).

Rare diseases often have an important impact on quality of life. Notwithstanding, PROMs were used as secondary endpoints in only 59% of the retrieved trials. Disease-specific HRQOL scales were included in 49% of RCTs. Cross-disease generic indicators were used in only 26% of the trials (Table 2). PROMs were more often included in the study design of Phase III trials and for autoimmune, metabolic, haematological, and oncological diseases (Table 2 and Supplementary Material, Table S1).

Results for oncology and oncohaematology present some peculiarities. RCTs are the predominant design in both areas (72% in oncology; 70.4% in oncohaematology), but open-label studies are more common—particularly in oncohaematology (79%)—due to the difficulty in blinding parenteral treatments, as was stated before. Active comparators are widely used (95% in oncohaematology; 56% in oncology). Surrogate endpoints are prevalent (60% in oncology; 85% in oncohaematology), while overall survival is more frequently used as an endpoint in oncology than in other fields (36%). Disease-specific PROMs are included in 48% and 44% of oncology and oncohaematology trials respectively (a little lower of a percentage than for other medicines), whereas generic PROMs were rarely used in oncology trials.

## 4. Discussion

This paper illustrates the design of clinical trials of medicines for rare indications that are expected to be launched by 2026 in Italy and whether it has been improving from an HTA perspective. Italy was used as a case study, but the analysis of trials is applicable to any HTA process.

### 4.1. Trial Phase and Design

The majority of studies are located in Phase III. This result was not expected, since drugs with an orphan designation (77% of the total) can be approved after Phase II, with conditional approval that requires post-marketing data collection to confirm the risk–benefit of the drug. Most of the trials are designed as RCTs and an active comparator was found in 46% of RCTs (47% for orphan drugs). The former result is particularly surprising: previous evidence shows a prevalence of single-arm trials. Head-to-head trials and the demonstration of an additional benefit are expected of a new drug for an indication in which an approved drug with an orphan drug status already exists. Double-blind design was also prevalent (61% of RCTs). The increase in Phase III and RCTs is an important step towards a better understanding of this evidence by HTA authorities but could also be interpreted in another (negative) way: since gaining reimbursement for drugs for ultra-rare diseases is challenging, the industry might have become discouraged to develop drugs for diseases in which Phase III and RCTs are unfeasible. We do not have data to confirm this hypothesis, since we do not have information on whether clinical development was not implemented or interrupted for ultra-rare diseases. We found that Phase III and RCTs accounted for 77% (data not reported in Supplementary Material) and 54% (Supplementary Material, Table S1) of all trials for ultra-rare diseases, respectively.

The use of placebos in RCTs, observed in 52% of cases, raises relevant ethical considerations. According to the current version of the Declaration of Helsinki, placebo-controlled groups should not be used when a suitable, approved treatment exists and can serve as an active comparator. However, our study design did not allow us to verify the availability or appropriateness of therapeutic alternatives at the time of trial initiation. As this information was not consistently available in public sources, we could not assess whether placebo use was ethically justified in each case. Nevertheless, this issue is particularly relevant in rare diseases, where therapeutic options may be limited or evolving, and deserves careful consideration in future research and regulatory discussions.

### 4.2. Endpoints

Surrogate indicators are used as the primary endpoint in the majority of trials and PROMs were included among the secondary endpoints in 59% of studies. The use of clinical endpoints different from overall survival as primary endpoints is quite common: overall survival is not the ideal endpoint, from the trial design perspective, when the effects of drugs on mortality can be detected only in the long term (i.e., when the disease does not have a poor prognosis). However, the use of clinical endpoints could be critical for rare diseases, since their surrogacy could be very uncertain when the trial is initiated.

It should also be acknowledged that the distinction between surrogate endpoints and other clinically relevant, non-surrogate outcomes is often blurred. As highlighted in the recent literature, defining and validating surrogacy is complex and context-dependent, especially for chronic diseases not associated with reduced life expectancy. In these cases, relevant endpoints may be misclassified or underestimated in terms of their clinical value, potentially disadvantaging certain therapeutic areas in HTA. While this analysis did not aim to reclassify endpoints beyond their reported definition, we recognise the importance of this debate and the need for further methodological work in this direction [27].

### 4.3. PROMs

PROMs were not included in 40.9% of clinical trials, with an unexpected higher proportion in oncology, were there is a very high number of available, disease-specific PROMs [28]. Disease-specific PROMs were more frequent (49.3%) than generic ones (25.9%). On the one hand, the scarce use of overall survival as the primary endpoint and generic PROMs are challenging for cost-effectiveness analysis. Extrapolations to detect the final impact on overall survival and HRQOL (to measure quality-adjusted life years—QALYs) are complex to manage. Secondary data are needed to detect the impact of disease progression on HRQOL to estimate the total effects on QALYs. On the other hand, generic PROMs are often not validated for rare diseases.

### 4.4. Limitations

The study has some limitations.

It may have been affected by selection bias since only Phase III trials were retrieved if the marketing authorisation dossier was not submitted to EMA/FDA (14% of the total considered in this analysis). The rationale for this choice was that the study was focused on pivotal studies for drugs expected to be launched by 2026 and no information was available about the intention to submit the marketing authorisation dossier after Phase II.

We did not carry out any comparison between pivotal studies for rare and non-rare indications. For sure, this would have enriched the discussion on the information gaps for rare disease medicines at market launch. However, our findings were informative enough, regardless this comparation, and would have required retrieving trials for non-rare diseases sufficiently similar to the detected rare disease trials to make an unbiased comparison.

We have not investigated other elements that could be interesting for assessors and payers, but were not available in public sources, including the number of the recruited patients and the length of the trials.

Finally, we have not scrutinised the surrogacy of clinical endpoints. Our aim was mainly descriptive: endpoint surrogacy and validity would have required a more profound analysis that was not foreseen in our research protocol and would have needed a structured consultation with clinicians and other experts.

## 5. Conclusions

Despite their limitations, our findings have at least two important policy implications. Our analysis shows an important improvement in clinical trial design for orphan drugs, compared to previous results from the perspective of HTA organisations [16,17]. Phase II, single-arm, and open-label trials could be considered acceptable for approval, but they are often criticised by HTA authorities and payers, the main drivers of the downgrading of clinical studies. Our study shows a great effort to strengthen the evidence for drugs for rare diseases. This could, in the future, accelerate the comparative assessment of drugs for rare diseases in Italy until 2028 and afterwards through the HTA EU regulation, thus making prospectively easier the interaction between the industry and the assessor/payers.

It is not clear whether this effort to prolong clinical development to Phase III and to strengthen clinical trial design is reducing the willingness to invest in rare diseases. For sure, if this was the case, it would be very important to understand for which drugs this effort is imperative and when single-arm and Phase II trials can be considered sufficient from a payer’s perspective (e.g., for ultra-rare diseases).

Notwithstanding, there is still room for improvement, in particular the incorporation of the patient’s perspective in the evaluation process. The use of HRQOL general scales should be enhanced to populate cost–utility studies with primary data. Disease-specific HRQOL questionnaires and PROMs are much more commonly used in clinical trials, but their role is widely acknowledged across countries and their validation should be better scrutinized.

## Figures and Tables

**Table 1:** Characteristics of the retrieved rare drugs.

	**#**	**%**		**#**	**%**
**Total**	**154**	**100.0%**	**Total**	**154**	**100.0%**
**Target**			**Rare vs** **. ultra-rare**		
Haematology	32	20.8%	Rare	141	91.6%
Onco-haematology	27	17.5%	Ultra rare	13	8.4%
Oncology	25	16.2%			
Metabolism	23	14.9%	**Orphan designation**		
Autoimmune diseases	22	14.3%	Yes	119	77.3%
Neurology	8	5.2%	No	35	22.7%
Endocrine system	7	4.5%			
Dermatology	3	1.9%	**Accelerated programmes** *		
Cardiovascular diseases	3	1.9%	Breakthrough	50	27.8%
Renal diseases	2	1.3%	Fast Track	43	23.9%
Ophthalmology	1	0.6%	PRIME	18	10.0%
Infections	1	0.6%	None	69	38.3%
**Biologic vs** **. NME**					
Biologic	78	50.6%			
NME	65	42.2%			
Non-NME	11	7.1%			

Rare drugs expected to be launched in Italy by 2026. * The number of observations is higher than the total, since a drug/indication can be included in more than one accelerated approval programme at the same time.

**Table 2:** Characteristics of clinical trials.

	Total		Oncology		Oncohaematology		Others	
	**#**	**%**	**#**	**%**	**#**	**%**	**#**	**%**
	**154**	**100.00%**	**25**	**16.3%**	**27**	**17.5%**	**102**	**66.2%**
**Trial phase**								
Phase III	124	80.5%	18	72.0%	20	74.1%	86	84.3%
Phase II/III	12	7.8%	1	4.0%	1	3.7%	10	9.80%
Phase II	16	10.4%	6	24.0%	4	14.8%	16	15.7%
Phase I/II	2	1.3%	0	0.0%	2	7.4%	0	0.0%
**Trial design**								
RCT	99	64.3%	18	72.0%	19	70.4%	62	60.8%
Single-arm	55	35.7%	7	28.0%	8	29.6%	40	39.2%
**Blindness (RCT)**								
Double-blind RCT	60	60.6%	8	44.4%	4	21.1%	48	77.4%
Open-label RCT	39	39.4%	10	55.6%	15	78.9%	14	22.6%
**Control arm (RCT)**								
Active comparator	46	46.0%	10	55.6%	18	94.7%	18	29.0%
Placebo	51	52.0%	8	44.4%	1	5.3%	42	67.8%
Not specified	2	2.0%	0	0.0%	0	0.0%	2	3.2%
**Primary endpoint**								
Surrogate endpoint	126	81.8%	15	60.0%	23	85.2%	89	87.2%
Overall Survival	15	9.7%	9	36.0%	4	14.8%	2	3.0%
Safety	9	5.8%	1	4.0%	0	0.0%	8	7.9%
Avoided hospitalization	4	2.6%	0	0.0%	0	0.0%	4	3.9%
**PROMS**								
No PROMs	63	40.9%	12	48.0%	14	51.9%	37	36.3%
Disease-specific PROMs	51	33.1%	10	40.0%	5	18.5%	36	35.3%
Both PROMs	25	16.2%	2	8.0%	7	25.9%	16	15.7%
Generic PROMs	15	9.8%	1	4.0%	1	3.7%	13	12.7%

Rare drugs expected to launch in Italy by 2026.

## Data Availability

The datasets used and/or analysed during the current study are available from the corresponding author on reasonable request.

## Supplementary Materials

The following supporting information can be downloaded at https://www.mdpi.com/article/10.3390/jmahp13030037/s1: Table S1 illustrates all detailed results of the analysis.

## Abbreviations

AIFA	Italian Medicines Agency (Agenzia Italiana del Farmaco)
DALY	Disability-Adjusted Life Years
GRADE	Grading of Recommendations Assessment, Development, and Evaluation
HRQOL	Health-Related Quality of Life
HTA	Health Technology Assessment
ICER	Incremental Cost–Effectiveness Ratio
NDA	New Drug Approval
NME	New Molecular Entity
PRIME	PRIority MEdicines
P&R	Price and Reimbursement
PRO(M)s	Patient-Reported Outcome (Measure)s
QALY	Quality-Adjusted Life Years
RCT	Randomized Control Trial
