# Supplementary material for: Pivotal Studies for Drugs About to Be Launched for Rare Diseases: Will They Better Support Health Technology Assessment and Market Access than in the Past?
Source: J Mark Access Health Policy. 2025 Jul 25;13(3):37. doi: 10.3390/jmahp13030037 (PMC12372023; doi:10.3390/jmahp13030037)
Supplement: Supplementary file 1 [file jmahp-13-00037-s001.zip › jmahp-3665255-supplementary.pdf]

Supplementary material

Table S1: Detailed result

**Trial Phase**		
	RCT	Single arm
Phase III (124)	69%	31%
Others (30)	47%	53%
	Double-blind	Open-label
Phase III (85)	56%	44%
Others (14)	86%	14%
	Active comparator	Placebo / Not-specified
Phase III (85)	51%	49%
Others (14)	21%	79%
	Primary endpoint: OS	Primary endpoint: others
Phase III (124)	12%	88%
Others (30)	0%	100%
	PROMS	No PROMS

Phase III (124)	65%	35%	
Others (30)	37%	63%	
	Disease-specific PROMS	Both PROMS	Generic PROMS
Phase III (124)	57%	28%	15%
Others (30)	46%	27%	27%

**Therapeutic area**			
	RCT	Single Arm	
Haematology (32)	44%	56%	
Oncohaematology (27)	70%	30%	
Oncology (25)	72%	28%	
Metabolism (23)	57%	43%	
Immunology (22)	73%	27%	
Neurology (8)	63%	37%	
Endocrin system (7)	86%	14%	
Others (10)	80%	20%	
	Double blind	Open-label	
Haematology (14)	79%	21%	
Oncohaematology (19)	21%	79%	
Oncology (18)	44%	56%	

Metabolism (13)	77%	23%	
Immunology (16)	69%	31%	
Neurology (5)	80%	20%	
Endocrine system (6)	83%	17%	
Others (8)	87%	13%	
	Active comparator	Placebo / Not specified	
Haematology (14)	14%	86%	
Oncohaematology (19)	95%	5%	
Oncology (18)	56%	44%	
Metabolism (13)	46%	54%	
Immunology (16)	37%	63%	
Neurology (5)	20%	80%	
Endocrine system (6)	17%	83%	
Others (8)	25%	75%	
	Primary endpoint: OS	Primary endpoint: others	
Haematology (32)	0%	100%	
Oncohaematology (27)	15%	85%	
Oncology (25)	36%	64%	

Metabolism (23)	4%	96%	
Immunology (22)	5%	95%	
Neurology (8)	0%	100%	
Endocrine system (7)	0%	100%	
Others (10)	0%	100%	
	PROMS	No PROMS	
Haematology (32)	62%	38%	
Oncohaematology (27)	48%	52%	
Oncology (25)	52%	48%	
Metabolism (23)	65%	35%	
Immunology (22)	82%	18%	
Neurology (8)	50%	50%	
Endocrine system (7)	29%	71%	
Others (10)	60%	40%	
	Disease-specific PROMS	Both PROMS	Generic PROMS
Haematology (20)	30%	45%	25%
Oncohaematology (13)	38%	54%	8%
Oncology (13)	77%	15%	8%
Metabolism (15)	67%	13%	20%
Immunology (18)	72%	11%	17%
Neurology (4)	50%	25%	25%

Endocrine system (2)	50%	50%	0%
Others (6)	66%	17%	17%

**Biologic vs non-biologic**		
	RCT	Single arm
Biologic (78)	55%	45%
Non-biologic (76)	74%	26%
	Double-blind	Open-label
Biologic (43)	42%	58%
Non-biologic (56)	75%	25%
	Active comparator	Placebo / Not-specified
Biologic (43)	60%	40%
Non-biologic (56)	36%	64%
	Primary endpoint: OS	Primary endpoint: others
Biologic (78)	15%	85%
Non-biologic (76)	4%	96%

	PROMS	No PROMS	
Biologic (78)	65%	35%	
Non-biologic (76)	53%	47%	
	Disease-specific PROMS	Both PROMS	Generic PROMS
Biologic (51)	61%	29%	10%
Non-biologic (40)	50%	25%	25%

**Rare vs ultra-rare**			
	RCT	Single arm	
Rare (141)	65%	35%	
Ultra-rare (13)	54%	46%	
	Double-blind	Open-label	
Rare (92)	60%	40%	
Ultra-rare (7)	71%	29%	
	Active comparator	Placebo / Not-specified	
Rare (92)	46%	54%	
Ultra-rare (7)	57%	43%	

	Primary endpoint: OS	Primary endpoint: others	
Rare (141)	11%	89%	
Ultra-rare (13)	0%	100%	
	PROMS	No PROMS	
Rare (141)	46%	54%	
Ultra-rare (13)	60%	40%	
	Disease-specific PROMS	Both PROMS	Generic PROMS
Rare (85)	54%	28%	18%
Ultra-rare (6)	83%	17%	0%

Orphan vs non-orphan			
	RCT	Single arm	
Orphan (119)	62%	38%	
Non-orphan (35)	71%	29%	
	Double-blind	Open-label	
Orphan (74)	64%	36%	
Non-orphan (25)	52%	48%	
	Active comparator	Placebo / Not-specified	
Orphan (74)	47%	53%	
Non-orphan (25)	44%	56%	
	Primary endpoint: OS	Primary endpoint: others	
Orphan (119)	8%	92%	
Non-orphan (35)	17%	83%	
	PROMS	No PROMS	
Orphan (119)	57%	43%	
Non-orphan (35)	66%	34%	

	Disease-specific PROMS	Both PROMS	Generic PROMS
Orphan (68)	52%	29%	19%
Non-orphan (23)	70%	21%	9%

**Accelerated approval programs**		
	RCT	Single Arm
Breakthrough (50)	54%	46%
Fast Track (43)	63%	37%
PRIME (18)	50%	50%
None (69)	75%	25%
	Double blind	Open-label
Breakthrough (27)	59%	41%
Fast Track (27)	74%	26%
PRIME (9)	56%	44%
None (52)	56%	44%
	Active comparator	Placebo / Not specified
Breakthrough (27)	48%	52%
Fast Track (27)	48%	52%

PRIME (9)	78%	22%	
None (52)	40%	50%	
	Primary endpoint: OS	Primary endpoint: others	
Breakthrough (50)	2%	98%	
Fast Track (43)	0%	100%	
PRIME (18)	0%	100%	
None (69)	20%	80%	
	PROMS	No PROMS	
Breakthrough (50)	58%	42%	
Fast Track (43)	56%	44%	
PRIME (18)	56%	44%	
None (69)	62%	38%	
	Disease-specific PROMS	Both PROMS	Generic PROMS
Breakthrough (50)	48%	28%	24%
Fast Track (43)	42%	42%	16%
PRIME (18)	50%	30%	20%
None (69)	70%	19%	11%
